# Acceptance and verification of the Halcyon‐Eclipse linear accelerator‐treatment planning system without 3D water scanning system

**DOI:** 10.1002/acm2.12719

**Published:** 2019-09-25

**Authors:** Song Gao, Tucker Netherton, Mikhail A. Chetvertkov, Yuting Li, Laurence E. Court, William E. Simon, Jie Shi, Peter A. Balter

**Affiliations:** ^1^ Department of Radiation Physics The University of Texas MD Anderson Cancer Center Houston TX USA; ^2^ Sun Nuclear Corporation 3275 Suntree Boulevard Melbourne FL 32940 USA; ^3^ Present address: Radiation Oncology Allegheny General Hospital 320 E. North Ave Pittsburgh 15212 USA; ^4^ Present address: Department of Radiation Oncology The Ohio State University Wexner Medical Center 460 W. 10th Ave Columbus OH 43210 USA

**Keywords:** acceptance and commissioning, Halcyon linear accelerator, ionization chamber array

## Abstract

We tested whether an ionization chamber array (ICA) and a one‐dimensional water scanner (1DS) could be used instead of a three‐dimensional water scanning system (3DWS) for acceptance testing and commissioning verification of the Varian Halcyon–Eclipse Treatment Planning System (TPS). The Halcyon linear accelerator has a single 6‐MV flattening‐filter‐free beam and a nonadjustable beam model for the TPS. Beam data were measured with a 1DS, ICA, ionization chambers, and electrometer. Acceptance testing and commissioning were done simultaneously by comparing the measured data with TPS‐calculated percent‐depth‐dose (PDD) and profiles. The ICA was used to measure profiles of various field sizes (10‐, 20‐, and 28 cm^2^) at depths of d_max_ (1.3 cm), 5‐, 10‐, and 20 cm. The 1DS was used for output factors (OFs) and PDDs. OFs were measured with 1DS for various fields (2–28 cm^2^) at a source‐to‐surface distance of 90 cm. All measured data were compared with TPS‐calculations. Profiles, off‐axis ratios (OAR), PDDs and OFs were also measured with a 3DWS as a secondary check. Profiles between the ICA and TPS (ICA and 3DWS) at various depths across the fields indicated that the maximum discrepancies in high‐dose and low‐dose tail were within 2% and 3%, respectively, and the maximum distance‐to‐agreement in the penumbra region was <3 mm. The largest OAR differences between ICA and TPS (ICA and 3DWS) values were 0.23% (−0.25%) for a 28 × 28 cm^2^ field, and the largest point‐by‐point PDD differences between 1DS and TPS (1DS and 3DWS) were −0.41% ± 0.12% (−0.32% ± 0.17%) across the fields. Both OAR and PDD showed the beam energy is well matched to the TPS model. The average ratios of 1DS‐measured OFs to the TPS (1DS to 3DWS) values were 1.000 ± 0.002 (0.999 ± 0.003). The Halcyon–Eclipse system can be accepted and commissioned without the need for a 3DWS.

## 1 INTRODUCTION

The newly designed Halcyon linear accelerator (Linac; Varian Medical Systems, Palo Alto, CA) has a single‐energy 6‐MV flattening filter‐free photon beam with maximum field size of 28 × 28 cm^2^ at the isocenter. The gantry is enclosed and the machine does not have isocenter lasers or a light field but does have external setup lasers outside of the bore. The external lasers are used for initial patient (or phantom) setup outside the bore, and the patient can be loaded to the isocenter position through predefined couch shifts from the initial position to the isocenter position. Orthogonal MV image pairs or MV cone‐beam computed tomography provides image‐guided position adjustment for actual treatment. The Halcyon Linac (version 1.0) has only MV imaging capabilities, and subsequent to this work, version 2.0 was released with kVp imaging.

The single‐energy Halcyon Linac is tightly matched with a treatment planning system (TPS) beam model that is predefined by Varian. The universal beam model in the Eclipse TPS for all Halcyon Linacs cannot be modified by the user and the TPS has no adjustable beam‐modeling tools. The Halcyon Linac and TPS were originally accepted and verified as a single package. Because of this, commissioning verification was based on AAPM Medical Physics Practice Guideline for Commissioning and QA of External Beam Planning Systems (MPPG5.a).^1^

We evaluated an acceptance testing and commissioning verification — the beam characteristics of the Halcyon Linac without need a 3D water scanning system (3DWS), and provided Halcyon users an efficient and low cost solution to clinically verify the machine/TPS system. The beam profiles for various field sizes and depths were measured with an ionization chamber array (IC PROFILER, Sun Nuclear Corporation, Melbourne, FL). Percent depth dose (PDD) data were scanned with a one‐dimensional water scanner (1DS). Output factors and absolute TG‐51 calibration were also obtained with the 1DS and ionization chambers and electrometer. TG‐51 dosimetry for the Halcyon was recently studied by Lloyd et al.^2^ with a 1DS, therefore it is not covered extensively in this work. In the current study, the output factors, PDDs, and profiles were compared with those calculated by the TPS. As a secondary validation, we also compared PDDs, output factors, and profiles with those measured with the 3DWS.

The purpose of this study was to evaluate whether an ionization chamber array (ICA) and a 1DS can be used for acceptance testing and commissioning beam data verification of the Varian Halcyon‐Eclipse TPS system without the need for a 3DWS system, and to establish appropriate guidelines for the acceptance and validation process for Halcyon–Eclipse linac‐TPS system.

## 2 METHODS

The pieces of equipment we studied for acceptance and commissioning verification of beam data for the Halcyon Linac are the following: an ICA (IC PROFILER) with a 1DS (1D SCANNER) with Sun Nuclear Corporation dosimetry software; a PC electrometer with 2‐channel calibration; and waterproof ion chambers (SNC125c and SNC600c).

### 2.1 Profile measurement

Beam profiles were measured with the IC PROFILER for various field sizes (10 × 10 cm^2^, 20 × 20 cm^2^, 28 × 28 cm^2^) and depths (d_max_, 5 cm, 10 cm, and 20 cm) at 90 cm source to surface distance (SSD). This ICA has 251 ion chamber detectors aligned along four profile axes: the principal x and y axes and two diagonal axes, with a 0.9 cm water equivalent inherent buildup from the top surface to the detectors. We used additional solid water buildup on top of the ICA to produce the depths for profile measurements. This ICA has 2.3 cm water equivalent inherent backscatter material which is sufficient for the data collection to compare with 3D water scans.^4^

**Figure 1:**
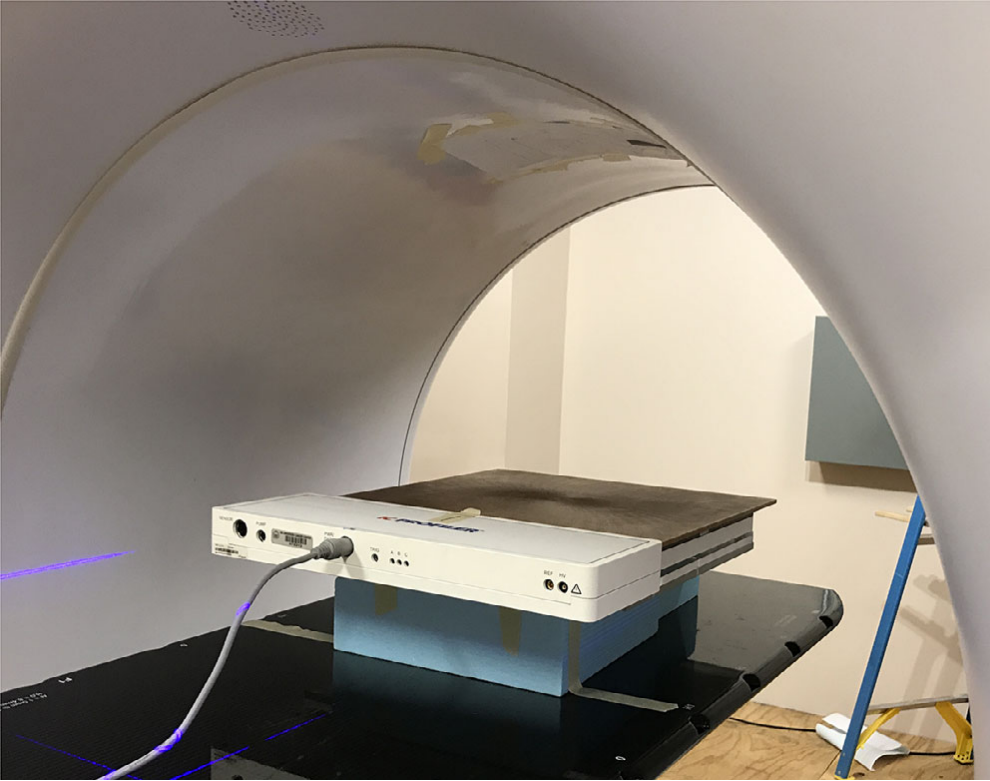
The IC array measurement setup position with solid water buildup.

Given the absence of light field and radiation isocenter lasers in this system, the ICA setup was done in three steps: (a) initial alignment with the outside bore lasers (virtual isocenter), followed by loading to the radiation isocenter; (b) image guidance for final alignment with two orthogonal MV images, using a lateral image to find the surface of the device, followed by determining the SSD and using an anterior‐posterior image to align the center of the ICA to the central axis (CAX) and to look for in‐plane rotations of the device; (c) acquiring test profiles to verify and fine‐tune, at the sub mm level, the beam CAX to the center of ICA by verifying the beam center along all four axes of the ICA. In steps (a) and (b), the ICA position was adjusted with couch shifts. The final positions for measurements with solid water buildup and with quad wedge plate are illustrated in Fig. 1. It should be noted that to achieve 90 cm SSD at d_max_ depth a foam block was added under the ICA due to limitations in the maximum couch height.

Before profile measurements were obtained, the detectors in the ICA were normalized using the manufacturer’s calibration procedure.^3^ This was done on the Halcyon Linac with appropriate thicknesses of buildup to match the measurement depths with each depth being saved as a separate calibration file. An extended 110 cm SSD and 28 × 28 cm^2^ field was used to ensure that all detectors used in later measurements were in the calibration field. The accuracy of the array calibration was evaluated according to previously proposed procedures,^4^ and the calibration uncertainties were <0.5% for all detectors in the field across the three different calibrations with different buildup.

To evaluate the beam profiles acquired by the ICA, all measured points in the high‐dose region, penumbra, and low‐dose tail regions were compared with the TPS calculations at various depths as recommended by MPPG5.a^1^ for primary evaluation. As a secondary validation, the profiles measured with ICA were also compared with those scanned previously with a 3DWS (IBA Blue Phantom 2). The 3DWS water tank setup procedure is as follows:^5^ We used the IBA Blue2 water tank for scanning measurements since it was the only system we had that could fit into the bore. In this system, the water tanks and other quality assurance devices cannot be aligned with lasers, light field, or mechanical devices at the isocenter; rather, the user must align the device to an external setup position via lasers and shift the device to isocenter, and then use image guidance to set the SSD and align the chamber. A procedure in the Halcyon “Instructions for Use” manual that involves portal images taken at oblique angles can be used to setup the tank. The location and alignment of the chamber can be visualized at depth by delivering 25‐50 MU using an anterior‐posterior image. A brass cap placed on the ionization chamber can be used to increase image contrast.

To obtain different dose regions of the profiles (high‐dose region, penumbra, and low‐dose tail regions) as recommended by MPPG5.a,^1^ the normalized and centered TPS‐calculated profile data with 1.0‐mm point spacing were exported to Microsoft Excel^®^, and steps taken as follows:^5^ (a) the first derivatives of all profiles were calculated and then normalized to 1.0; (b) points on either side of the first derivative full width at half maximum indicate the penumbra region, and (c) points outside this FWHM region specify the low dose tail and high dose regions. Thus, the TPS profiles served to define the beam regions so that measured profiles could be compared based on MPPG5.a.

The profiles from ICA data, TPS calculations in water phantom, and 3DWS data were exported to Microsoft Excel^®^. All profiles were normalized to the central of axis and sampled to 0.5 cm point spacing; the point‐by‐point intensity differences (%) between ICA and TPS (ICA and 3DWS) values were calculated at the location of the ICA detectors (0.5 cm detector spacing) in the high dose and low‐dose tail regions. The average and standard deviation for all the points in high‐dose region were calculated, whereas the low dose region only the maximum point‐by‐point differences are reported. Distance to agreement (DTA) in penumbra region between the ICA profiles and those from TPS (ICA and 3DWS) values was calculated for different field sizes at various depths.

The off‐axis ratio (OAR), defined as the ratio of the average measurements at a fixed distance (e.g., 80% of the field size) along the profiles in two orthogonal axes from the beam CAX to the measurement at the CAX, is used as the beam energy metric.^6^ Recent studies indicated that an OAR‐based metric is more sensitive to energy changes than a PDD metric.^6^, ^7^ We also compared the OAR on the principal x and y axes from the profiles measured with ICA to those from TPS calculations and from 3DWS profiles.

### 2.2 Percent depth dose

**Figure 2:**
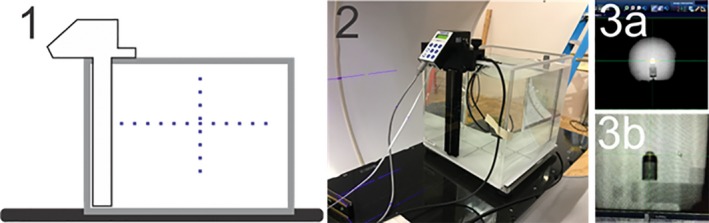
1DS water tank setup. 1: water tank is aligned at external setup position. 2: water tank is filled, and position calibrated. 3: Image guidance is performed to set SSD and visualize the chamber (3a without brass cap; 3b with brass cap). 1DS, one‐dimensional water scanner.

PDD scans were obtained in a 1DS water tank with the SNC125c waterproof ion chambers and PC Electrometer. The 1D water tank was set up in the same manner as the 3D water tank (Fig. 2), and has enough clearance to fit inside the bore due to its smaller size. If the procedure described in the Halcyon user manual is used to obtain an alignment for a 90 cm SSD for all measurements, this SSD setup procedure can be verified by first setting the SSD to 100 cm by using orthogonal imaging and then lowering the table by 10 cm. The depth was set as the effective point‐of‐measurement for the chamber. The scanning speed we used was continuous at 0.25cm/s and the scanning depth ranged from 0 to 30 cm.

PDDs were measured with 1DS for various field sizes (2 × 2 cm^2^, 4 × 4 cm^2^, 6 × 6 cm^2^, 8 × 8 cm^2^, 10 × 10 cm^2^, 20 × 20 cm^2^, and 28 × 28 cm^2^) and then compared with the TPS (1DS and 3DWS) values. For all scanned PDDs (via the 1DS and 3DWS), the data were smoothed and renormalized to 100% by the values at d_max_ depth for each field size. All PDDs values (1DS, TPS, and 3DWS) were sampled at 1‐mm point spacing. The point‐by‐point differences of PDDs between the 1DS measurements and the TPS calculations and 3DWS scans were calculated for the various field sizes. The average, standard deviation (σ), the minimum and maximum PDD differences for all the points from 1.0 cm to 30.0 cm depth range were also calculated.

### 2.3 Output factors and dose calibration

Like the PDD, the output factors were measured in the 1DS with the SNC125c waterproof ion chambers and PC electrometer, correcting for the effective point of measurement. The measurement was done at the depth of 10 cm with an SSD of 90 cm for a range of field sizes (from 2 × 2 cm^2^ through 28 × 28 cm^2^) and normalized to the corresponding values at the 10 × 10 cm^2^ field size. TG‐51 calibrations were done in the same 1DS with a 0.6‐cc waterproof Farmer chamber (PTW 30013, Freiburg, Germany) and PC electrometer. The TG51 protocol was followed to calibrate 1.00 cGy/MU to water at depth of maximum dose and SSD of 100 cm. The TG51 addendum (specifying procedures for flattening filter‐free beams) was used to determine the beam quality conversion factor k_Q_ and P_rp_, the correction factor that accounts for off‐axis variation in the beam profile.^2^, ^8^ The 1DS measured data were compared to both output factors from TPS and 3DWS values.

## 3 RESULTS

### 3.1 Profile comparison

**Figure 3:**
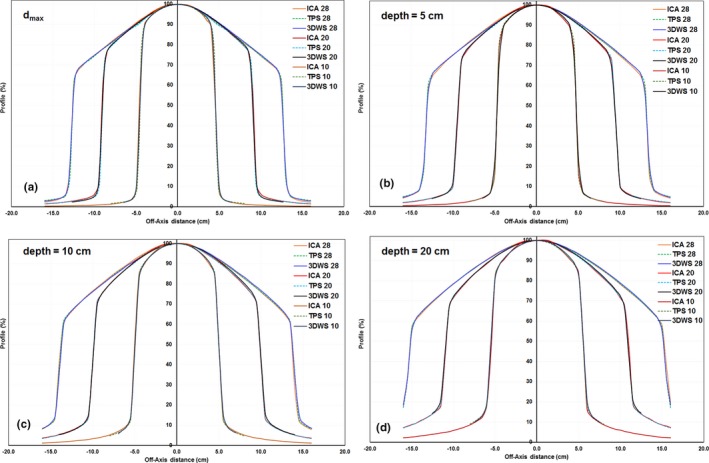
Profile measured with an ionization chamber profiler (ICA) with an SSD of 90 cm, for 10 × 10, 20 × 20, and 28 × 28 cm^2^ fields compare to TPS calculated and 3DWS scanned profiles of the same setup and field. Depths: (a) d_max_, (b) 5 cm, (c) 10 cm, and (d) 20 cm. 3DWS, 3D water scanning system; TPS, treatment planning system; SSD, source to surface distance.

Beam profiles were measured with the ICA at different d_max_, 5 cm, 10 cm, and 20 cm depths along in‐plane, cross‐plane, and diagonal directions at 90 cm SSD for 10 × 10 cm^2^, 20 × 20 cm^2^, and 28 × 28 cm^2^ field sizes. The profiles were normalized to the corresponding central axis value for each field size (Fig. 3).

Discrepancies between ICA and TPS (ICA and 3DWS) values were quantified by using the mean, standard deviation (σ), the minimum (min), and maximum (max) intensity differences of all points in the high‐dose region (Table 1). All of the points in the high‐dose region indicate that the differences between ICA and TPS (ICA and 3DWS) were within 1.2% (1.8%). These data agree within the 2.0% criteria recommend in MPPG5.a.

**Table 1:** High‐dose region profile comparison between the ICA and TPS (ICA and 3DWS) for three fields and four depths. Shaded cells are mean (average) ± *SD* (σ) of point‐by‐point differences (%) from profiles. Clear cells are the maximum (max) and minimum (min) difference values.

	Depth, cm		10 × 10 cm^2^	20 × 20 cm^2^	28 × 28 cm^2^
ICA‐TPS	d_max_	mean±σ	−0.03 ± 0.24	0.21 ± 0.32	0.19 ± 0.39
		max/min	0.4/–0.8	1.1/−0.5	1.2/–1.0
	5	mean±σ	−0.24 ± 0.37	−0.08 ± 0.41	−0.06 ± 0.45
		max/min	0.3/–1.0	0.7/–1.0	0.8/–1.4
	10	mean±σ	−0.1 ± 0.36	−0.04 ± 0.29	0.06 ± 0.24
		max/min	0.6/–1.1	0.6/–0.8	0.7/–0.8
	20	mean±σ	0.05 ± 0.46	0.01 ± 0.25	−0.08 ± 0.20
		max/min	1.0/–1.2	0.7/−0.6	0.5/‐0.8
ICA‐3DWS	d_max_	mean±σ	−0.06 ± 0.38	−0.09 ± 0.27	−0.09 ± 0.33
		max/min	0.9/–1.0	0.4/−0.8	0.6/–0.7
	5	mean±σ	−0.19 ± 0.37	–0.14 ± 0.37	−0.21 ± 0.45
		max/min	0.5/–1.0	1.1/−1.1	0.6/–1.2
	10	mean±σ	0.09 ± 0.53	–0.19 ± 0.47	−0.05 ± 0.31
		max/min	1.5/–0.7	1.4/–1.1	0.8/–0.8
	20	mean±σ	0.07 ± 0.67	−0.15 ± 0.45	−0.17 ± 0.40
		max/min	1.8/–0.9	1.6/−1.1	1.8/–0.8

Abbreviations: 3DWS, 3D water tanks scans; ICA, ionization chamber array; TPS, treatment planning system.

The maximum point‐by‐point difference in the low‐dose tail region profiles between ICA and TPS (ICA and 3DWS) values were less than 3%. The maximum DTA difference in the penumbra region between ICA and TPS (ICA and 3DWS) values was less than 3 mm. These differences conform to the recommend in MPPG5.a for all fields in the test at various depths (Table 2). For the profiles measured with the ICA, our results demonstrated that the ICA‐measured profiles matched very well with TPS and 3DWS data in high‐dose, low‐dose tail, and penumbra regions. Note that Table 2 presents the maximum differences from ICA and TPS (ICA and 3DWS), but the point locations are not coincident, they are determined by the maximum.

**Table 2:** Low‐dose region profiles and penumbra region comparison between the ICA and TPS (ICA and 3DWS) for three fields and four depths. Shaded cells are maximum point‐by‐point differences (%) from profiles. Clear cells are the maximum DTA in the penumbra region.

	Depth, cm		10 × 10 cm^2^	20 × 20 cm^2^	28 × 28 cm^2^
ICA‐TPS	d_max_	Low dose, %	1.0	1.8	1.7
		DTA, mm	–1.25	2.44	2.82
	5	Low dose, %	2.5	−0.6	−2.8
		DTA, mm	–2.18	−2.91	−2.73
	10	Low dose, %	1.3	−0.65	1.0
		DTA, mm	−1.68	1.75	−1.06
	20	Low dose, %	−1.11	1.1	2.1
		DTA, mm	−2.36	1.71	2.46
ICA‐3DWS	d_max_	Low dose, %	−1.6	2.2	1.2
		DTA, mm	1.9	−2.46	1.56
	5	Low dose, %	−2.8	−1.6	−2.7
		DTA, mm	2.94	2.2	−2.72
	10	Low dose. %	−2.1	−2.21	1.7
		DTA, mm	−2.94	1.54	−2.2
	20	Low dose, %	−2.3	−2.6	−2.8
		DTA, mm	2.75	−1.74	1.34

Abbreviations: 3DWS, 3D water tanks scans; DTA, Distance to agreement; ICA, ionization chamber array; TPS, Treatment Planning System.

The ICA measured OARs at approximately 80% of the field size from CAX on the orthogonal x and y axes for fields 20 × 20 cm^2^ and 28 × 28 cm^2^ at depths d_max_ and 10 cm showed excellent agreement with both the TPS and 3DWS, within 0.4% and 0.3%, respectively (Table 3).

**Table 3:** Differences in off‐axis ratio between measurements between ICA and TPS (ICA and 3DWS).

Field size	20 × 20 cm^2^		28 × 28 cm^2^	
Depth, cm	d_max_	10	d_max_	10
ICA‐TPS	−0.02%	−0.38%	0.23%	0.04%
ICA‐3DWS	0.25%	0.25%	−0.25%	−0.22%

Abbreviations: 3DWS, 3D water tanks scans; ICA, ionization chamber array; TPS, treatment planning system.

### 3.2 Percent depth dose

**Figure 4:**
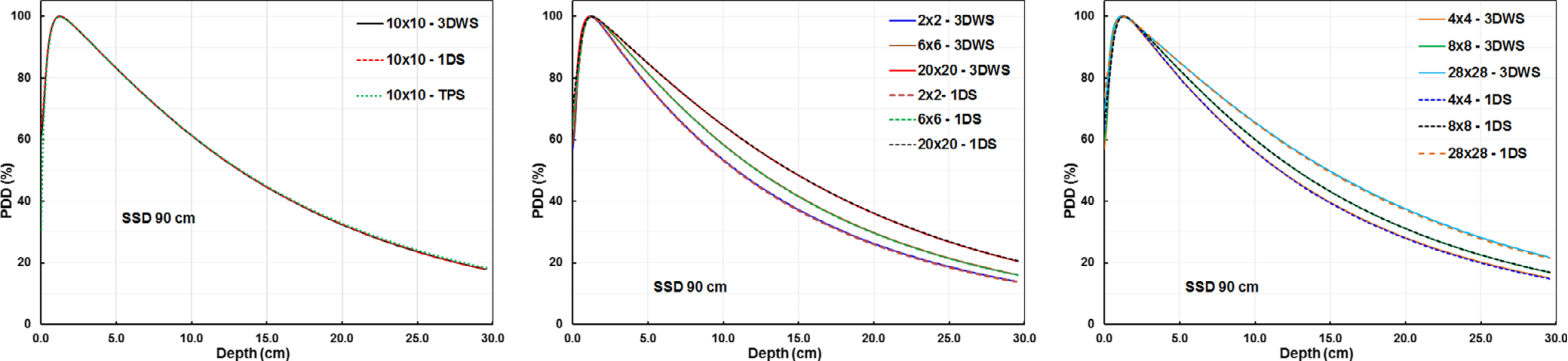
PDDs scanned using the 1DS water tank with an SSD of 90 cm for field sizes from 2 × 2 cm^2^ to 28 × 28 cm^2^ compared with PDDs of the same setup calculated by the treatment planning system (TPS) and 3D water tanks scans (3DWS). 1DS, one‐dimensional water scanner; PDDs, percent‐depth‐dose; SSD, source to surface distance.

The PDD data measured for seven different field sizes with the 1DS were compared with TPS data and 3DWS measured data. The PDDs (1DS, TPS, and 3DWS) data were exported into Excel and qualitatively compared by plotting them on the same graph. We found that the shapes of the PDD curves of these three methods were consistent (Fig. 4). Quantitatively, the average (mean) ± *SD* (σ) with the minimum (min) and maximum (max) differences of the PDDs between 1DS and TPS (1DS and 3DWS) for all the points from 1.0 cm to 30.0 cm depth range with the various field sizes were compared. The results indicated that the differences between 1DS versus the TPS and 3DWS were within 0.8% for all points, and the mean ± σ values were within 0.5% (Table 4). The values of d_max_ for three different methods of each field size were also presented, the differences of d_max_ among three methods are within 0.8 mm.

**Table 4:** PDD comparison between the 1DS and TPS (1DS and 3DWS) from 1.0 cm to 30.0 cm for seven fields. Shaded cells are mean (average) ± *SD* (σ) of point‐by‐point differences (%) between PDDs. Clear cells are the maximum (max) and minimum (min) difference values.

	Field	2 × 2 cm^2^	4 × 4 cm^2^	6 × 6 cm^2^	8 × 8 cm^2^	10 × 10 cm^2^	20 × 20 cm^2^	28 × 28 cm^2^
1DS‐TPS	mean ± σ	−0.41 ± 0.12	−0.30 ± 0.11	−0.29 ± 0.17	−0.25 ± 0.27	−0.16 ± 0.22	0.02 ± 0.24	−0.25 ± 0.23
	max/min	0.13/−0.59	0.15/−0.49	0.14/−0.47	0.29/−0.56	0.23/−0.57	0.80/−0.32	0.77/−0.50
1DS‐3DWS	mean ± σ	−0.25 ± 0.10	−0.05 ± 0.13	−0.05 ± 0.09	−0.05 ± 0.10	0.04 ± 0.09	−0.03 ± 0.08	−0.32 ± 0.17
	max/min	0.13/−0.43	0.43/−0.25	0.30/−0.19	0.30/−0.23	0.35/−0.18	0.25/−0.18	0.14/−0.55
d_max_ (cm)	1DS	1.19	1.29	1.26	1.29	1.25	1.2	1.06
	TPS	1.15	1.25	1.25	1.20	1.20	1.18	1.12
	3DWS	1.16	1.26	1.30	1.25	1.26	1.26	1.06

Abbreviations: 3DWS, 3D water tanks scans; 1DS, one‐dimensional water scanner; PDDs, percent‐depth‐dose; TPS, treatment planning system.

### 3.3 Output factors

The 1DS measured output factors ranged from 0.801 to 1.111, and the results were compared with those calculated by the TPS and measured using the 3DWS (Table 5).

**Table 5:** The output factors measured using the 1DS with an SSD of 90 cm at a depth of 10 cm for various field sizes and compared with data from the TPS and measurements obtained with a 3DWS.

Field Size, cm^2^	Output factor			Difference, ratio	
	1DS	TPS	3DWS	1DS/TPS	1DS/3DWS
2 × 2	0.801	0.801	0.803	1.000	0.998
4 × 4	0.881	0.878	0.880	1.003	1.001
6 × 6	0.931	0.927	0.931	1.004	1.000
8 × 8	0.970	0.970	0.970	1.000	1.000
10 × 10	1.000	1.000	1.000	1.000	1.000
14 × 14	1.044	1.045	1.045	0.999	0.999
20 × 20	1.085	1.088	1.088	0.997	0.997
24 × 24	1.102	1.106	1.107	0.996	0.995
28 × 28	1.111	1.115	1.117	0.998	0.995
4 × 20	0.940	0.941	0.938	0.999	1.002
20 × 4	0.940	0.940	0.940	1.000	1.000

Abbreviations: 3DWS, 3D water tanks scans; 1DS, one‐dimensional water scanner; SSD, source to surface distance; TPS, treatment planning system.

For all field sizes, the mean (±σ) normalized output factors from the 1DS to the TPS was 1.000 ± 0.002, with a maximum of 0.4% and minimum of −0.4%. Similarly, for 1DS values normalized to 3DWS values, the mean (±σ) was 0.999 ± 0.003, with a maximum of 0.3% and minimum of −0.5%. 1DS measured data matched very well with TPS and 3DWS data.

## 4 DISCUSSIONS

For accurate profile measurements with an ICA, the array calibration accuracy is essential to avoid systematic errors. We recommend that the same thickness of solid water buildup be used for both the array calibration and for the actual beam measurement. The array calibration should be validated before the measurements and this can be done with any stable beam of a similar energy.^4^ We noticed that the out‐of‐field (OOF) profiles measured with the ICA were higher than those measured in the 3DWS and those calculated by the TPS, especially at deeper depths. The over‐response is likely due to a change in the response of the detector to the low‐energy OOF scatter. As part of this work the manufacturer developed a correction method, not yet commercially released, for the OOF detector responses. Applying the OOF correction parameter to the ICA data, and comparing them with the TPS and 3DWS data, the differences against TPS (and 3DWS) for majority out of field data points were <1%, and the maximum discrepancies in the low‐dose tail region were <3% for all fields in the test at various depths (see Table 2) which meet the recommendation of MPPG5.a.

A 3DWS is traditionally used for acquiring the data for TPS commissioning. The Halcyon Linac is provided with an associated Eclipse TPS model that is universal to all such machines. Since this changes the task from modeling the TPS to accepting the standard model. The data collection at the time of acceptance/commissioning can be restricted to the data need to verify rather than to create the TPS model. We have demonstrated that these data can be acquired using a qualified 2D ion chamber detector array and a 1DS water scanning system. This is a considerably more efficient and lower‐cost solution than the use of a 3DWS. Our findings showed that with proper ICA array calibration and setup procedures, the 1DS combine with ICA can sufficiently verify Halcyon system characteristics as being within the desired specifications for patient treatment, and can achieve the same quality as beam data acquired with a 3DWS. Likewise, other types of 1D water scanners with beam scanning capability and 2D detector arrays can also be used for these measurements when these devices demonstrate performance similar to our finding.

The measurements of the beam profiles with ICA were done for field sizes larger than 10 × 10 cm^2^. It should be sufficient for acceptance and verification of the beam model since we were not commissioning the beam. For beams with small field size, we recommend relying on the end‐to‐end tests and intensity‐modulated radiation therapy (IMRT) quality assurance (QA) tests.^5^ The resolution of beam profile is limited by the detector spacing in ICA, and may not be able to acquire accurate beam profile data for small field size. In the very recent study, Karimnia et al., improved the resolution of beam profile data measured with ICA by moving the ICA relative to the CAX with fractional distances of the detector spacing to acquire multiple measurements of a single radiation field size and then reconstruct all of the measured data.^9^ Their results demonstrated that with this technique, the beam profiles measured with ICA have comparable data quality to 3DWS data.

The PDD profiles scanned with 1DS agreed very well to TPS and 3DWS data for the field sizes other than smallest 2 × 2 cm^2^ field and largest 28 × 28 cm^2^ field (see Fig. 4). The smallest field has largest measurement uncertainty, for the largest field, some loss in scatter happen in 1DS, but the maximum difference between 1DS and TPS (1DS and 3DWS) was within 0.8% (0.5%).

We also noticed that the output factors measured using the 1DS were lower than those measured in 3DWS for field sizes greater than 20 × 20 cm^2^. This may indicate some loss in scatter in 1DS, but the maximum difference of the 1DS to 3DWS ratios was 0.5% and is within the 2% tolerance level^1^ for this parameter in MPPG5.a and should be sufficient for validating an existing beam model.

This work was restricted to the Halcyon but could be applied to any delivery system with a predetermined beam model in the TPS including the use at centers that are accepting new machines against an existing beam model. Of course this is most advantageous for machines such as the Halcyon or other treatment units that have geometries that make the setup of traditional 3DWS difficult.

## 5 CONCLUSIONS

We demonstrated that a 2D detector array and 1D water scanner system can be used for acceptance and clinical verification of the Halcyon‐Eclipse TPS static beam data, and the resulting beam data match those of the TPS and a 3DWS. Use of the ICA/1DS greatly speeds up the acceptance and validation process and reduces effort by eliminating the need for a 3D water tank.

## CONFLICT OF INTEREST

This work (SG) was supported in part by a grant from Sun Nuclear Corporation.
